# The crystal structure of a new polymorph of hexa­aqua­nickel(II) bis­(6-oxo-1,6-di­hydro­pyridine-3-carboxyl­ate)

**DOI:** 10.1107/S2056989015022422

**Published:** 2015-11-28

**Authors:** Rubén Pérez-Aguirre, Sonia Pérez-Yáñez, Garikoitz Beobide, Oscar Castillo, Juan Manuel Gutiérrez-Zorrilla, Antonio Luque

**Affiliations:** a Departamento de Química Inorgánica., Facultad de Ciencia y Tecnología, Universidad del País Vasco, UPV/EHU, Apdo. 644, E-48080 Bilbao, Spain

**Keywords:** crystal structure, polymorph, 6-oxo-1,6-di­hydro­pyridine-3-carboxyl­ate anion, hydrogen bonding,

## Abstract

In a new polymorph of the title salt, [Ni(H_2_O)_6_](C_6_H_4_NO_3_)_2_, the metal atom of the cationic complex lies on a symmetry centre and is coordinated by six water mol­ecules to provide a quite regular octa­hedral coordination environment. These cations inter­act with 6-oxo-1,6-di­hydro­pyridine-3-carboxyl­ate anions through electrostatic inter­actions and by means of O—H⋯O and N—H⋯O hydrogen bonds involving the carboxyl­ate, keto and protonated imine groups of the anion, and the coordinating water mol­ecules from the cationic complex entity to generate a supra­molecular three-dimensional architecture. The previously reported polymorph of this compound presents a network of hydrogen bonds, in which the organic anions establish mutual hydrogen-bonding inter­actions involving their keto and protonated imine groups.

## Related literature   

The zinc and cobalt analogues (Zhang *et al.*, 2005 ▸; Song *et al.*, 2005 ▸; Zhang & Ng, 2005*a* ▸) of the title salt are isostructural with the previously reported polymorph of [Ni(H_2_O)_6_](C_6_H_4_NO_3_)_2_ (Zhang & Ng, 2005*b* ▸). It is worth mentioning that although the authors claimed a lactim tautomer of the organic anion to be present in all these structures, the C—O bond length seems to indicate of a lactam tautomer as in the case of the title compound. For additional examples of coordination complexes with 6-oxo-1,6-di­hydro­pyridine-3-carboxyl­ate anions and copper(II), see: Zeng *et al.* (2007 ▸).
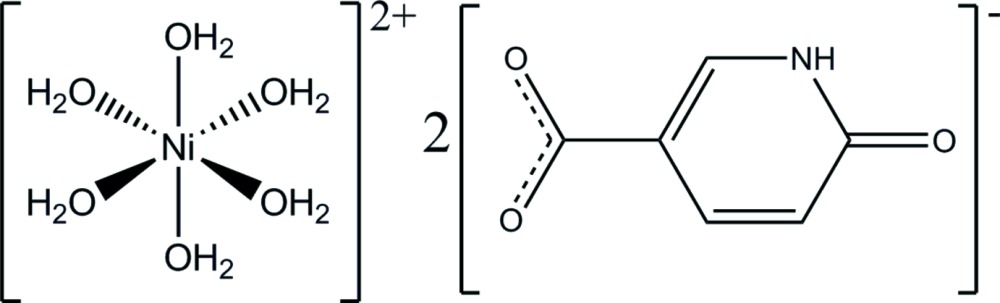


## Experimental   

### Crystal data  

[Ni(H_2_O)_6_](C_6_H_4_NO_3_)_2_
*M* *_r_* = 443.01
Triclinic, 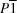

*a* = 6.2620 (5) Å
*b* = 7.1053 (7) Å
*c* = 10.7101 (10) Å
α = 102.461 (8)°
β = 96.754 (7)°
γ = 114.823 (8)°
*V* = 410.49 (7) Å^3^
*Z* = 1
Mo *K*α radiation
μ = 1.26 mm^−1^
*T* = 100 K
0.08 × 0.07 × 0.06 mm

### Data collection  

Bruker SMART 1K CCD area-detector diffractometer
Absorption correction: analytical (*CrysAlis RED*; Oxford Diffraction, 2003 ▸) *T*_min_ = 0.888, *T*_max_ = 0.936
2781 measured reflections
1801 independent reflections
1654 reflections with *I* > 2σ(*I*)
*R*_int_ = 0.020

### Refinement  

*R*[*F* ^2^ > 2σ(*F* ^2^)] = 0.032
*wR*(*F* ^2^) = 0.070
*S* = 1.06
1801 reflections
146 parameters
H atoms treated by a mixture of independent and constrained refinement
Δρ_max_ = 0.45 e Å^−3^
Δρ_min_ = −0.39 e Å^−3^

### 

Data collection: *CrysAlis PRO* (Agilent, 2012 ▸); cell refinement: *CrysAlis PRO*; data reduction: *CrysAlis PRO*; program(s) used to solve structure: *SIR92* (Altomare *et al.*, 1993 ▸); program(s) used to refine structure: *SHELXL97* (Sheldrick, 2008 ▸); molecular graphics: *ORTEP-3 for Windows* (Farrugia, 2012 ▸); software used to prepare material for publication: *WinGX* (Farrugia, 2012 ▸).

## Supplementary Material

Crystal structure: contains datablock(s) I, global. DOI: 10.1107/S2056989015022422/wm5241sup1.cif

Structure factors: contains datablock(s) I. DOI: 10.1107/S2056989015022422/wm5241Isup2.hkl

**Fig. 1.**
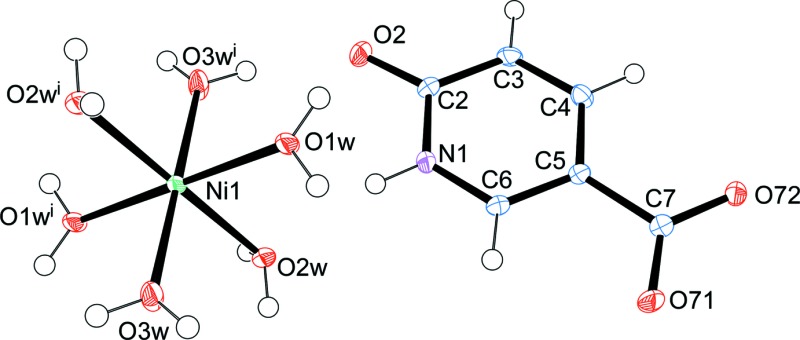
The structures of the molecular entities in (I), drawn with displacement ellipsoids at the 50% probability level. [Symmetry code: −x + 1, −y, −z + 1.]

Supporting information file. DOI: 10.1107/S2056989015022422/wm5241Isup3.cdx

x y z . DOI: 10.1107/S2056989015022422/wm5241fig1.tif
The structures of the mol­ecular entities in (I), drawn with displacement ellipsoids at the 50% probability level. [Symmetry code: −*x* + 1, −*y*, −*z* + 1.]

**Fig. 2.**
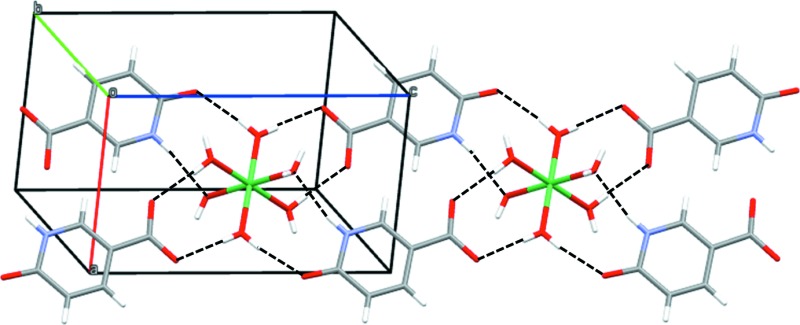
Hydrogen-bonding interactions (dashed lines) taking place between the [Ni(H2O)6]2+ complex cations and the 6-oxo-1,6-dihydropyridine-3-carboxylate anions.

2 6 2+ . DOI: 10.1107/S2056989015022422/wm5241fig2.tif
Hydrogen-bonding inter­actions (dashed lines) taking place between the [Ni(H_2_O)_6_]^2+^ complex cations and the 6-oxo-1,6-di­hydro­pyridine-3-carboxyl­ate anions.

CCDC reference: 1438522

Additional supporting information:  crystallographic information; 3D view; checkCIF report

## Figures and Tables

**Table 1:** Selected bond lengths (Å)

Ni1—O1*W*	2.0184 (16)
Ni1—O3*W*	2.0242 (16)
Ni1—O2*W*	2.0990 (16)

**Table 2:** Hydrogen-bond geometry (Å, °)

*D*—H⋯*A*	*D*—H	H⋯*A*	*D*⋯*A*	*D*—H⋯*A*
N1—H1⋯O2*W*	0.87 (2)	2.06 (3)	2.906 (2)	167 (2)
O1*W*—H11*W*⋯O71^i^	0.77 (3)	1.85 (3)	2.612 (2)	173 (3)
O1*W*—H12*W*⋯O72^ii^	0.78 (3)	1.97 (3)	2.748 (2)	173 (3)
O2*W*—H21*W*⋯O2^iii^	0.88 (3)	1.90 (3)	2.772 (2)	173 (2)
O2*W*—H22*W*⋯O2^iv^	0.77 (3)	1.98 (3)	2.743 (2)	169 (3)
O3*W*—H31*W*⋯O72^i^	0.74 (3)	1.92 (3)	2.660 (2)	174 (3)
O3*W*—H32*W*⋯O2^v^	0.81 (3)	2.01 (3)	2.813 (2)	172 (3)

Symmetry codes: (i) 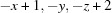; (ii) 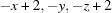; (iii) 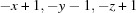; (iv) 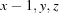; (v) 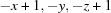.

## supplementary crystallographic information

### S1. Experimental

6-Oxo-1,6-dihydropyridine-3-carboxylic acid (0.8 mmol) and Ni(NO_3_)_2_·6H_2_O (0.4 mmol) were dissolved in 40 ml of distilled water. After stirring for half an hour, the solution was left evaporating at room temperature. Two weeks later light green crystals of the title compound were obtained.

### S2. Refinement

H atoms bonded to N and O atoms were located in a difference map and were refined with *U*_iso_(H) = 1.2*U*_eq_(N) and *U*_iso_(H) = 1.5*U*_eq_(O). Other H atoms were positioned geometrically and refined using a riding model with C—H = 0.93 Å and with *U*_iso_(H) = 1.2*U*_eq_(C).

### Crystal data

**Table:**

[Ni(H_2_O)_6_](C_6_H_4_NO_3_)_2_	*Z* = 1
*M**_r_* = 443.01	*F*(000) = 230
Triclinic, *P*1	*D*_x_ = 1.792 Mg m^−^^3^
*a* = 6.2620 (5) Å	Mo *K*α radiation, λ = 0.71073 Å
*b* = 7.1053 (7) Å	Cell parameters from 2781 reflections
*c* = 10.7101 (10) Å	θ = 2.0–28.2°
α = 102.461 (8)°	µ = 1.26 mm^−^^1^
β = 96.754 (7)°	*T* = 100 K
γ = 114.823 (8)°	Block, light green
*V* = 410.49 (7) Å^3^	0.08 × 0.07 × 0.06 mm

### Data collection

**Table:**

Bruker SMART 1K CCD area-detector diffractometer	1801 independent reflections
Radiation source: sealed tube	1654 reflections with *I* > 2σ(*I*)
Graphite monochromator	*R*_int_ = 0.020
Detector resolution: 8.192 pixels mm^-1^	θ_max_ = 28.2°, θ_min_ = 2.0°
thin–slice ω scans	*h* = −5→8
Absorption correction: analytical (*CrysAlis RED*; Oxford Diffraction, 2003)	*k* = −9→5
*T*_min_ = 0.888, *T*_max_ = 0.936	*l* = −14→13
2781 measured reflections	

### Refinement

**Table:**

Refinement on *F*^2^	Primary atom site location: structure-invariant direct methods
Least-squares matrix: full	Secondary atom site location: difference Fourier map
*R*[*F*^2^ > 2σ(*F*^2^)] = 0.032	Hydrogen site location: mixed
*wR*(*F*^2^) = 0.070	H atoms treated by a mixture of independent and constrained refinement
*S* = 1.06	*w* = 1/[σ^2^(*F*_o_^2^) + (0.0191*P*)^2^ + 0.3293*P*] where *P* = (*F*_o_^2^ + 2*F*_c_^2^)/3
1801 reflections	(Δ/σ)_max_ < 0.001
146 parameters	Δρ_max_ = 0.45 e Å^−^^3^
0 restraints	Δρ_min_ = −0.39 e Å^−^^3^

### Special details

**Table:**

Refinement. Refinement of *F*^2^ against ALL reflections. The weighted *R*-factor *wR* and goodness of fit *S* are based on *F*^2^, conventional *R*-factors *R* are based on *F*, with *F* set to zero for negative *F*^2^. The threshold expression of *F*^2^ > σ(*F*^2^) is used only for calculating *R*-factors(gt) *etc*. and is not relevant to the choice of reflections for refinement. *R*-factors based on *F*^2^ are statistically about twice as large as those based on *F*, and *R*- factors based on ALL data will be even larger.

### Fractional atomic coordinates and isotropic or equivalent isotropic displacement parameters (Å^2^)

**Table:**

	*x*	*y*	*z*	*U*_iso_*/*U*_eq_	
Ni1	0.5000	0.0000	0.5000	0.01281 (12)	
O1W	0.7185 (3)	0.1609 (3)	0.68193 (15)	0.0168 (3)	
O71	0.5271 (3)	−0.1565 (3)	1.13681 (15)	0.0202 (4)	
O2W	0.3038 (3)	−0.2654 (3)	0.56622 (15)	0.0154 (3)	
O3W	0.2933 (3)	0.1461 (3)	0.55076 (17)	0.0231 (4)	
O2	0.8636 (3)	−0.3362 (3)	0.62046 (14)	0.0176 (3)	
O72	0.8231 (3)	−0.2245 (3)	1.21937 (15)	0.0202 (4)	
N1	0.6616 (3)	−0.2558 (3)	0.76999 (18)	0.0162 (4)	
C2	0.8355 (4)	−0.3128 (4)	0.7379 (2)	0.0141 (4)	
C4	0.9331 (4)	−0.3021 (3)	0.9661 (2)	0.0140 (4)	
H4	1.0278	−0.3170	1.0333	0.017*	
C3	0.9726 (4)	−0.3378 (4)	0.8423 (2)	0.0148 (4)	
H3	1.0917	−0.3793	0.8264	0.018*	
C5	0.7512 (4)	−0.2432 (3)	0.9937 (2)	0.0130 (4)	
C7	0.6965 (4)	−0.2048 (3)	1.1271 (2)	0.0144 (4)	
C6	0.6196 (4)	−0.2203 (4)	0.8921 (2)	0.0154 (4)	
H6	0.4995	−0.1797	0.9070	0.018*	
H1	0.574 (4)	−0.240 (4)	0.708 (3)	0.018*	
H31W	0.266 (5)	0.177 (4)	0.615 (3)	0.023*	
H21W	0.262 (5)	−0.387 (5)	0.505 (3)	0.023*	
H22W	0.189 (5)	−0.270 (4)	0.588 (3)	0.023*	
H11W	0.650 (5)	0.171 (4)	0.736 (3)	0.023*	
H12W	0.844 (5)	0.170 (4)	0.712 (3)	0.023*	
H32W	0.236 (5)	0.196 (5)	0.503 (3)	0.038 (9)*	

### Atomic displacement parameters (Å^2^)

**Table:**

	*U*^11^	*U*^22^	*U*^33^	*U*^12^	*U*^13^	*U*^23^
Ni1	0.0108 (2)	0.0231 (2)	0.0093 (2)	0.01104 (17)	0.00397 (15)	0.00593 (16)
O1W	0.0119 (8)	0.0321 (10)	0.0095 (8)	0.0130 (7)	0.0037 (6)	0.0053 (7)
O71	0.0201 (8)	0.0353 (10)	0.0169 (8)	0.0204 (8)	0.0102 (7)	0.0109 (7)
O2W	0.0127 (8)	0.0239 (9)	0.0126 (8)	0.0102 (7)	0.0056 (6)	0.0058 (7)
O3W	0.0303 (10)	0.0467 (12)	0.0121 (8)	0.0320 (9)	0.0110 (7)	0.0135 (8)
O2	0.0198 (8)	0.0248 (9)	0.0126 (8)	0.0127 (7)	0.0073 (6)	0.0067 (7)
O72	0.0180 (8)	0.0384 (10)	0.0112 (8)	0.0187 (8)	0.0048 (6)	0.0079 (7)
N1	0.0152 (9)	0.0275 (11)	0.0121 (9)	0.0135 (8)	0.0047 (8)	0.0094 (8)
C2	0.0133 (10)	0.0176 (11)	0.0130 (10)	0.0074 (9)	0.0061 (8)	0.0054 (9)
C4	0.0132 (10)	0.0156 (11)	0.0138 (10)	0.0084 (9)	0.0010 (8)	0.0033 (9)
C3	0.0118 (10)	0.0190 (11)	0.0170 (11)	0.0102 (9)	0.0053 (8)	0.0042 (9)
C5	0.0113 (10)	0.0159 (11)	0.0115 (10)	0.0058 (8)	0.0038 (8)	0.0038 (8)
C7	0.0127 (10)	0.0169 (11)	0.0133 (11)	0.0062 (9)	0.0039 (8)	0.0047 (9)
C6	0.0130 (10)	0.0233 (12)	0.0145 (11)	0.0107 (9)	0.0066 (9)	0.0075 (9)

### Geometric parameters (Å, º)

**Table:**

Ni1—O1W	2.0184 (16)	O2—C2	1.275 (2)
Ni1—O1W^i^	2.0184 (16)	O72—C7	1.262 (3)
Ni1—O3W^i^	2.0242 (16)	N1—C6	1.356 (3)
Ni1—O3W	2.0242 (16)	N1—C2	1.366 (3)
Ni1—O2W^i^	2.0990 (16)	N1—H1	0.87 (2)
Ni1—O2W	2.0990 (16)	C2—C3	1.417 (3)
O1W—H11W	0.77 (3)	C4—C3	1.367 (3)
O1W—H12W	0.78 (3)	C4—C5	1.409 (3)
O71—C7	1.253 (2)	C4—H4	0.9300
O2W—H21W	0.88 (3)	C3—H3	0.9300
O2W—H22W	0.77 (3)	C5—C6	1.366 (3)
O3W—H31W	0.74 (3)	C5—C7	1.503 (3)
O3W—H32W	0.81 (3)	C6—H6	0.9300
			
O1W—Ni1—O1W^i^	180.0	H31W—O3W—H32W	106 (3)
O1W—Ni1—O2W	89.95 (6)	C6—N1—C2	124.28 (18)
O1W—Ni1—O2W^i^	90.05 (6)	C6—N1—H1	118.1 (16)
O1W—Ni1—O3W	88.06 (7)	C2—N1—H1	117.6 (16)
O1W—Ni1—O3W^i^	91.94 (7)	O2—C2—N1	118.94 (19)
O2W—Ni1—O3W	92.99 (7)	O2—C2—C3	125.81 (19)
O2W—Ni1—O3W^i^	87.01 (7)	N1—C2—C3	115.24 (18)
O1W^i^—Ni1—O3W^i^	88.06 (7)	C3—C4—C5	121.08 (19)
O1W^i^—Ni1—O3W	91.94 (7)	C3—C4—H4	119.5
O3W^i^—Ni1—O3W	180.0	C5—C4—H4	119.5
O1W^i^—Ni1—O2W^i^	89.95 (6)	C4—C3—C2	121.22 (19)
O3W^i^—Ni1—O2W^i^	92.99 (7)	C4—C3—H3	119.4
O1W^i^—Ni1—O2W	90.05 (6)	C2—C3—H3	119.4
O3W^i^—Ni1—O2W	87.01 (7)	C6—C5—C4	117.18 (19)
O2W^i^—Ni1—O2W	180.0	C6—C5—C7	119.20 (18)
Ni1—O1W—H11W	114 (2)	C4—C5—C7	123.62 (18)
Ni1—O1W—H12W	130 (2)	O71—C7—O72	125.51 (19)
H11W—O1W—H12W	110 (3)	O71—C7—C5	116.72 (18)
Ni1—O2W—H21W	110.1 (17)	O72—C7—C5	117.77 (18)
Ni1—O2W—H22W	116 (2)	N1—C6—C5	120.98 (19)
H21W—O2W—H22W	108 (3)	N1—C6—H6	119.5
Ni1—O3W—H31W	129 (2)	C5—C6—H6	119.5
Ni1—O3W—H32W	124 (2)		
			
C6—N1—C2—O2	−178.1 (2)	C6—C5—C7—O71	1.2 (3)
C6—N1—C2—C3	1.1 (3)	C4—C5—C7—O71	−178.9 (2)
C5—C4—C3—C2	1.2 (3)	C6—C5—C7—O72	−179.1 (2)
O2—C2—C3—C4	177.9 (2)	C4—C5—C7—O72	0.8 (3)
N1—C2—C3—C4	−1.2 (3)	C2—N1—C6—C5	−1.0 (3)
C3—C4—C5—C6	−1.0 (3)	C4—C5—C6—N1	0.8 (3)
C3—C4—C5—C7	179.1 (2)	C7—C5—C6—N1	−179.3 (2)

Symmetry code: (i) −*x*+1, −*y*, −*z*+1.

### Hydrogen-bond geometry (Å, º)

**Table:**

*D*—H···*A*	*D*—H	H···*A*	*D*···*A*	*D*—H···*A*
N1—H1···O2*W*	0.87 (2)	2.06 (3)	2.906 (2)	167 (2)
O1*W*—H11*W*···O71^ii^	0.77 (3)	1.85 (3)	2.612 (2)	173 (3)
O1*W*—H12*W*···O72^iii^	0.78 (3)	1.97 (3)	2.748 (2)	173 (3)
O2*W*—H21*W*···O2^iv^	0.88 (3)	1.90 (3)	2.772 (2)	173 (2)
O2*W*—H22*W*···O2^v^	0.77 (3)	1.98 (3)	2.743 (2)	169 (3)
O3*W*—H31*W*···O72^ii^	0.74 (3)	1.92 (3)	2.660 (2)	174 (3)
O3*W*—H32*W*···O2^i^	0.81 (3)	2.01 (3)	2.813 (2)	172 (3)

Symmetry codes: (i) −*x*+1, −*y*, −*z*+1; (ii) −*x*+1, −*y*, −*z*+2; (iii) −*x*+2, −*y*, −*z*+2; (iv) −*x*+1, −*y*−1, −*z*+1; (v) *x*−1, *y*, *z*.
