# Supplementary figures and images for: The crystal structure of a new polymorph of hexa­aqua­nickel(II) bis­(6-oxo-1,6-di­hydro­pyridine-3-carboxyl­ate)
Source: Acta Crystallogr E Crystallogr Commun. 2015 Nov 28;71(Pt 12):m238–9. doi: 10.1107/S2056989015022422 (PMC4719852; doi:10.1107/S2056989015022422)

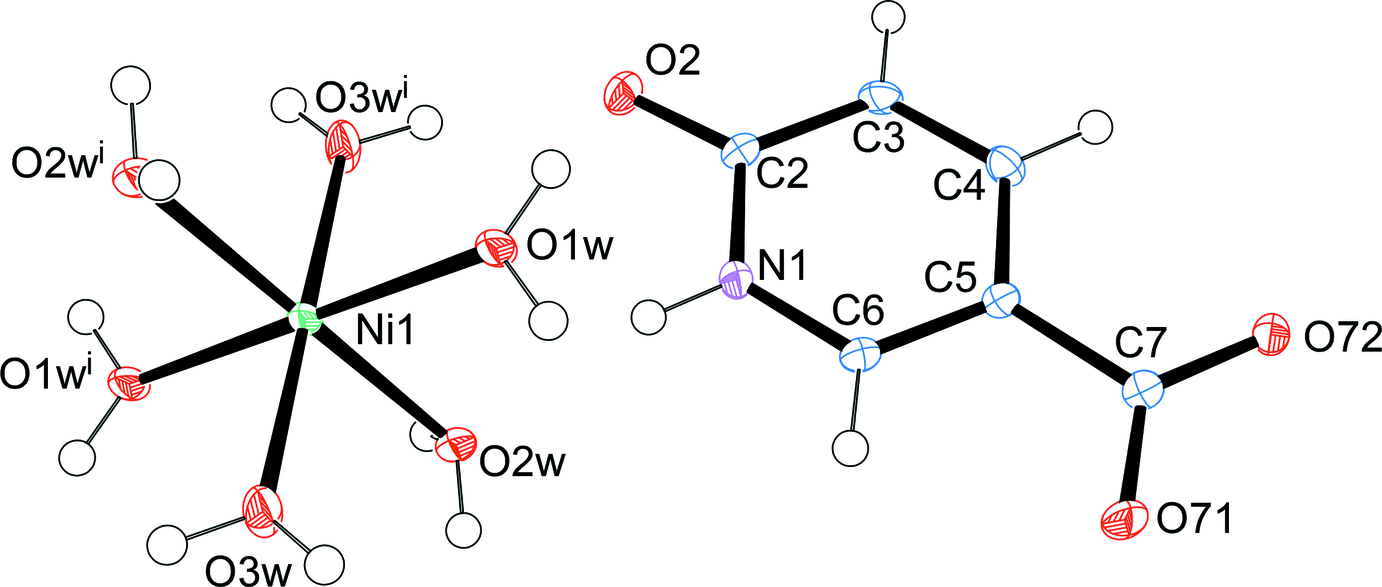

Supplement: Supplementary file 4 [file e-71-0m238-fig1.tif]

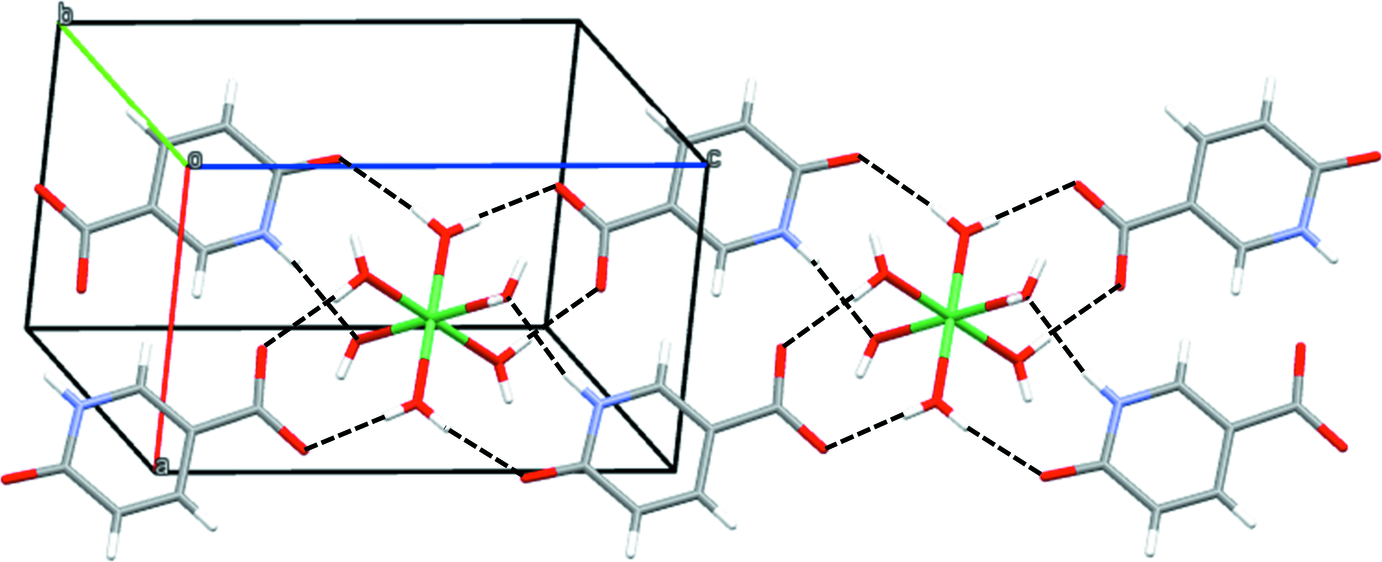

Supplement: Supplementary file 5 [file e-71-0m238-fig2.tif]
